# Long Chain *N*-acyl Homoserine Lactone Production by *Enterobacter* sp. Isolated from Human Tongue Surfaces

**DOI:** 10.3390/s121114307

**Published:** 2012-10-24

**Authors:** Wai-Fong Yin, Kathiravan Purmal, Shenyang Chin, Xin-Yue Chan, Kok-Gan Chan

**Affiliations:** 1 Division of Genetics and Molecular Biology, Institute of Biological Sciences, Faculty of Science, University of Malaya, 50603 Kuala Lumpur, Malaysia; E-Mails: yinwaifong@yahoo.com (W.-F.Y.); shenyang86@yahoo.com (S.C.); evepy88@yahoo.com (X.-Y.C.); 2 Department of General Dental Practice and Oral and Maxillofacial Imaging, Faculty of Dentistry, Universiti Sains Malaysia, 50603 Kuala Lumpur, Malaysia; E-Mail: drkathi@hotmail.com

**Keywords:** *N*-acylhomoserine lactone, *N*-dodecanoyl-L-homoserine lactone (C12-HSL), oral cavity, posterior dorsal surface, quorum sensing, tongue

## Abstract

We report the isolation of *N*-acyl homoserine lactone-producing *Enterobacter* sp. isolate T1-1 from the posterior dorsal surfaces of the tongue of a healthy individual. Spent supernatants extract from *Enterobacter* sp. isolate T1-1 activated the biosensor *Agrobacterium tumefaciens* NTL4(pZLR4), suggesting production of long chain AHLs by these isolates. High resolution mass spectrometry analysis of these extracts confirmed that *Enterobacter* sp. isolate T1-1 produced a long chain *N*-acyl homoserine lactone, namely *N*-dodecanoyl-homoserine lactone (C12-HSL). To the best of our knowledge, this is the first isolation of *Enterobacter* sp., strain T1-1 from the posterior dorsal surface of the human tongue and *N*-acyl homoserine lactones production by this bacterium.

## 1. Introduction

The human oral cavity is a rich source of microorganisms [1] where a dynamic interaction exists between the host environment and the oral bacteria consortium. Although the oral cavity consists of a complex microbial environment, Streptococcus is the major genus and is well studied [2]. Other oral bacteria identified include *Actinomyces* spp., *Capnocytophaga* spp., *Eikenella* spp., *Haemophilus* spp., *Prevotella* spp., *Propionibacterium* spp., and *Veillonella* spp. and *Fusobacterium* spp. [2]. These oral bacteria interact with the environment by attaching to surfaces and establishing mixed-species communities and this routinely requires cell-to-cell communication, which often results in the formation of biofilms. It has been suggested that oral bacterial species do not use *N*-acyl homoserine lactone (AHL)-based cell-to-cell signalling mechanisms [2–4], but instead, the autoinducer-2 (AI-2) signalling mechanism is commonly used by most oral bacteria, which include *Prevotella intermedia*, *Porphyromonas gingivalis*, *Streptococcus gordonii* and *Streptococcus mutans*[5–8].

AHLs are arguably the most studied quorum sensing (QS) signalling molecules in proteobacteria, and are produced by a LuxI synthase that will bind to LuxR protein [9]. When the concentration of these AHLs reaches the threshold level, the AHL-luxR complex will regulate a set of genes which occur in a population density-dependent manner, leading to population driven changes in several functions including virulence determinants, antibiotic production, bioluminescence, and biofilm formation [10–13]. QS bacteria have been isolated from various sources and habitats, including the human body [14–18].

Both the Gram-positive and Gram-negative bacteria have been implicated in several systemic infections [1]. Recently, we have isolated two AHL-producing *K. pneumoniae* strains from the posterior dorsal surface of the tongue of a healthy individual [17]. Here we report the isolation of *Enterobacter* sp. isolate T1-1capable of producing C12-HSL isolated from the tongue surfaces of a healthy individual, collectively this result provide evidence that oral bacteria are not limited to AI-2 activity.

## 2. Experimental Section

### 2.1. Bacterial Strains

*Agrobacterium tumefaciens* NTL4(pZLR4) [19] was used as biosensor and *Escherichia coli* DH5α served as a host for DNA manipulations. *A. tumefaciens* NTL4(pZLR4) was cultured in AB medium (containing 6% (w/v) K_2_HPO_4_, 2% (w/v) KH_2_PO_4_, 2% (w/v) NH_4_Cl, 0.6% (w/v) MgSO_4_·7H_2_0, 0.3% (w/v) KCl, 0.02% (w/v) CaCl_2_, and 0.005% (w/v) FeSO_4_·7H_2_O) or agar (solidified with 1.5% (w/v) bacto-agar), supplemented with 30 μg/mL gentamicin and 0.5% w/v glucose [19]. For AHL detection with *A. tumefaciens* NTL4(pZLR4), AB agar without gentamicin was supplemented with 20 μg/mL X-gal. *A. tumefaciens* NTL4(pZLR4) will cause a blue pigmentation on AB agar supplemented with X-gal in the presence of long chain AHLs. All other bacteria were cultured in Luria-Bertani (LB) medium (1% (w/v) tryptone, 0.5% (w/v) yeast extract, and 1% (w/v) NaCl), broth or agar (solidified with 1.5% (w/v) bacto-agar). All LB media were buffered with 50 mM 3-[*N-*morpholino]propanesulfonic acid (MOPS) to pH 5.5 to prevent opening of lactone ring of AHLs under alkali condition [20]. Where necessary, growth media were supplemented with 100 μg/mL ampicillin. *E. coli* cells and oral bacteria were grown at 37 °C whereas the biosensor strain was grown at 28 °C.

### 2.2. Isolation of Bacteria from Tongue Surface Debris

We have previously reported isolation of bacteria from oral orthodontics buccal tubes and AHL-producing bacterium from human oral cavity [17,21]. Here, tongue surface debris sample was collected in 2008 from an individual with healthy oral condition at the Faculty of Dentistry, University of Malaya. This study was approved by the Ethics Committee of the Faculty. In a previous report, we have isolated oral bacteria from tongue surface debris [17]. Pure colonies were obtained by several passages on the LB agar and screened for AHL production using biosensor *A. tumefaciens* NTL4(pZLR4). Of the bacteria screened, isolate T1-1 which activated *A. tumefaciens* NTL4(pZLR4) was selected for further analysis.

### 2.3. Strain Identification

All DNA extraction, purification, manipulations and PCR of 16S rDNA genes were carried out as previously described [14,22]. For PCR amplification of 16S rDNA genes from the genomic DNA, the universal primer pairs 27F and 1525R were used as described before [22]. Universal primers T7, SP6, and internal primers previously designed to anneal to internal target regions of the 16S rDNA were used [23]. LASERGENE software package (DNASTAR, US) was used to edit and analyse nucleotide sequences alignments. MEGA version 4.0 [24] was used for phylogenetic analysis and trees were generated using aligned 16S rDNA gene sequences with the Neighbour-Joining algorithm. Bootstrap analyses for 1,000 resamplings were used to ensure robustness and reliability of trees constructed.

### 2.4. Extraction and Detection of AHLs from Bacterial Culture Supernatants

In order to screen for oral bacteria that produces AHLs, oral bacteria were cross-streaked with the biosensor *A. tumefaciens* NTL4(pZLR4) [25] whereby a blue pigmentation on *A. tumefaciens* NTL4(pZLR4) lawn suggests the presence of long chain AHLs. Bacteria cells showed positive screening results were inoculated into 100 mL of LB broth and grown overnight. Overnight grown cells were adjusted to an OD_600_ of 1.0 and the spent supernatant was extracted twice with equal volume of acidified ethyl acetate (0.1% v/v acetate acid). The organic layer was collected in a separation funnel, dried over excessive amounts of anhydrous magnesium sulphate, filtered, and evaporated to dryness. Residues were dissolved in 100 μL of acetonitrile and stored at −20 °C. AHLs extract was further analysed by spotting 1 μL of the AHL extract onto the *A. tumefaciens* NTL4(pZLR4) lawn before sending for liquid chromatography mass spectrometry.

### 2.5. Mass Spectrometry (MS) Analysis of AHL

High resolution mass spectrometry was performed as previously described [17,26] using the Agilent RRLC 1200 system equiped with an Agilent ZORBAX Rapid Resolution HT column (100 mm × 2.1 mm, 1.8 μm particle size). Mobile phases A and B were 0.1% v/v formic acid in water and 0.1% v/v formic acid in acetonitrile, respectively. The gradient profile is as follow (time (min), mobile phase A: mobile phase B): 0 min, 60%:40%; 5 min, 20%:80%; 7–10 min, 5%:95%; 11–13 min, 60%:40%). The high resolution ESI-MS and ESI-MS/MS analysis was performed using an Agilent 6500 Q-TOF, operated in the ESI-positive mode, with probe capillary voltage fixed at 3,000 V; desolvation temperature of 350 °C; sheath gas set at 11 mL/hour; and nebulizer pressure of 50 psi. Nitrogen gas was used as the collision gas for the MS/MS analysis, with collision energy set at 20 eV. MS data was analysed by using Agilent MassHunter.

## 3. Results and Discussion

### 3.1. Enrichment and Characterization of Bacteria

**Figure 1.**
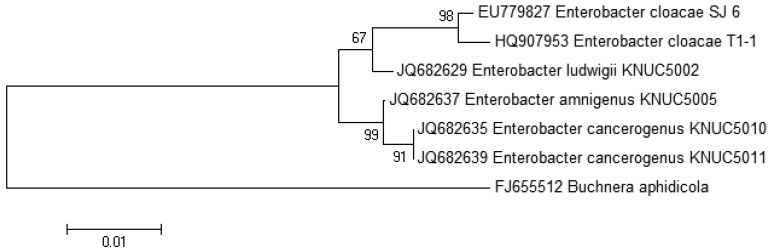
Phylogenetic analysis of oral bacterial isolate T1-1. 16S rDNA-based phylogenetic tree showing the phylogenetic position of isolate T1-1. The horizontal bar at the bottom represents evolutionary distance as number of changes per nucleotide position. *Buchnera aphidicola* was included as outgroup. GenBank accession numbers are given as number preceded the names of bacteria. Numbers at the branch nodes are bootstrap values (only those >50 are shown).

The growth medium became turbid at the first cycle (after 48 hour) and in the following cycles, suggesting that bacterial growth occurred during the enrichment procedure. Enrichment was in vitamin-free KG medium [16] containing C7-HSL. We use the comparatively rare odd number *N*-acyl side chain namely C7-HSL as previously reported [17]. For each enrichment cycle, cell culture was serially diluted and spread onto LB agar to obtain pure colonies. Bacterial colonies with distinctive morphologies were obtained after several successive streaks on LB agar and isolate T1-1 was selected for further analysis. *Enterobacter* sp. isolate T1-1 was molecularly identified by their 16S rDNA. Phylogenetic analysis of isolate T1-1 was performed by analyzing the partial sequence its 16S rDNA gene sequences (647 nucleotides), which has been deposited into GenBank, with accession number of HQ907953. Web-based search indicated T1-1 was *Enterobacter* sp. and further phylogenetic analysis confirmed that T1-1 was *Enterobacter* sp. closely related to the *E. cloacae* strain SJ6 (Figure 1).

### 3.2. Detection of AHLs by *Enterobacter* sp. Isolate T1-1

**Figure 2.**
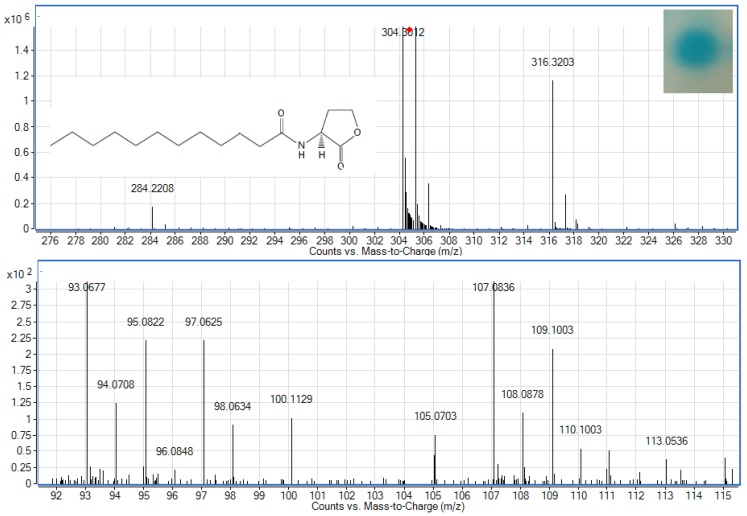
Detection of AHLs by *A. tumefaciens* NTL4(pZLR4) and mass spectrometry analysis of spent supernatants extracts of *Enterobacter* sp. (T1-1). Inset (top right) shows the AHL production of oral bacterial isolate *Enterobacter* sp. (T1-1) as detected by spotting the AHLs extract onto the biosensor lawn. Blue pigmentation showed that *Enterobacter* sp. isolate T1-1 produced long chain AHLs. Graph shows the mass spectra of parental ion of C12-HSL (*m/z* 284.2208, upper panel) and product ions of C12-HSL in MS-MS analysis (*m/z* 95.0822, 109.1003, lower panel).

To examine whether *Enterobacter* sp. isolate T1-1 produced AHLs, spent culture supernatant was extracted with acidified ethyl acetate (0.1% (v/v) acetate acid) and bioassay was used to detect AHLs in the concentrated extracts. *A. tumefaciens* NTL4(pZLR4) was used to detect AHLs in extracts obtained from the spent supernatant where the culture medium were acidic KG medium amended with MOPS to prevent lactonolysis by alkali. The biosensor was activated as evident by the blue pigmentation formed. In cross-streaked assay, oral bacterial isolate T1-1 activated the biosensor *A. tumefaciens* NTL4(pZLR4) suggested the production of long chain AHLs by isolate T1-1 (Figure 2, inset).

AHLs extracted from the spent supernatant of *Enterobacter* sp. isolate T1-1 was spotted onto the biosensor *A. tumefaciens* NTL4(pZLR4) lawn and a clear blue spot was observed (Figure 2). To further verify the presence of AHLs in the extracts, we used high resolution mass spectrometry that provides unequivocal determination of these AHL molecules in the spent supernatants. The mass spectra data indicated long chain AHLs have been detected. High resolution mass spectrometry analysis confirmed the presence of C12-HSL (*m/z* 284.2208) (Figure 2, upper panel) in the spent supernatant of T1-1 (*Enterobacter* sp. isolate T1-1). The ESI-MS/MS spectrum of C12-HSL shows fragments (*m/z* 95.0822, 109.1003) (Figure 2, lower panel) typical of a lactone-moiety [26]. Taken the biosensor and mass spectra data together, it is unequivocal confirming the presence of C12-HSL in the spent supernatant of *Enterobacter* sp. (T1-1).

It has been reported that *Enterobacter aerogenes*, *K. pneumoniae* and *K. oxytoca* all show AHL production, but these isolates only produce AHLs when grown microaerophilically in LB medium [27]. Interestingly, *Enterobacter* sp. isolate T1-1 isolated in the present work showed AHL production when grown aerobically in low-pH defined LB medium. Similar to our previous finding that reports the production of AHL by *K. pneumoniae* from oral cavity [17], here our result shows the quorum sensing activity of *Enterobacter* sp. isolate T1-1. Acid tolerance test indicated that *Enterobacter* sp. isolate T1-1 was viable when grown for 24 h in LB buffered at pH 5 remained viable at pH 3 (data not shown). Since QS regulates many important phenotypes including virulence production, hence study on QS in oral bacteria may lead to better understanding of how these oral bacteria colonise oral cavity. Natural products isolated from plants that show anti-QS activity may provide solution to prevent oral infection caused by QS pathogens [28].

In this study, *Enterobacter* sp. isolate T1-1 was isolated from the posterior dorsal surface of the tongue. In a study of the oral prevalence of aerobic and facultatively anaerobic Gram-negative rods and yeasts, it is reported that the oral prevalence of these microorganisms are 41.7% and Enterobacteriaceae species represents 73% of all these isolates. The most commonly found species are *E. cloacae* and *K. pneumoniae*[29]. Our finding on the isolation of strain T1-1 is in agreement with this report [29] but this is the first report of AHL-producing *Enterobacter* sp. isolated from the human tongue surface. In contrast, several genera of oral bacteria have been reported to produce AI-2, synthesized by LuxS [2,3,5,7]. Our work has expand the QS research in oral bacteria from AI-2 to AHL, currently we are performing whole genome sequencing of this bacterium to study the AHL synthase genes in *Enterobacter* sp. isolate T1-1.

## 4. Conclusions/Outlook

This work is the first documentation of AHL-producing ***Enterobacter*** sp. isolated from the human tongue surface. This enables us to have a deeper understanding on the oral bacterial communication and might be providing useful information for the AHL-based quorum sensing mechanism in human oral cavity.
